# *Apis mellifera syriaca* Venom: Evaluation of Its Anticoagulant Effect, Proteolytic Activity, and Cytotoxicity along with Its Two Main Compounds—MEL and PLA2—On HeLa Cancer Cells

**DOI:** 10.3390/molecules27051653

**Published:** 2022-03-02

**Authors:** Carole Yaacoub, Rim Wehbe, Yahya Salma, Dany El-Obeid, Romeo El Bersaoui, Bruno Coutard, Ziad Fajloun

**Affiliations:** 1 Laboratory of Applied Biotechnology (LBA3B), Azm Center for Research in Biotechnology and Its Applications, EDST, Lebanese University, Tripoli 1300, Lebanon; carole.yaacoub@etu.univ-amu.fr (C.Y.); yahya_salma@hotmail.com (Y.S.); 2 Unité des Virus Émergents, Aix-Marseille University-IRD 190-Inserm 1207, IHU Méditerranée Infection, 13005 Marseille, France; bruno.coutard@univ-amu.fr; 3 Biology Department, Faculty of Arts and Sciences, American University of Beirut, Beirut 1107 2020, Lebanon; rimg.wehbe@gmail.com; 4 Department of Biology, Faculty of Sciences 3, Lebanese University, Michel Slayman Tripoli Campus Ras Maska, Tripoli 1352, Lebanon; rbersawi@ul.edu.lb; 5 Faculty of Agriculture and Veterinary Sciences, Lebanese University, Dekwaneh, Beirut 2832, Lebanon; dany.elobeid@ul.edu.lb

**Keywords:** bee venom, *Apis mellifera syriaca*, anticoagulant effect, proteolytic activity, HeLa cell lines, melittin, PLA2

## Abstract

Bee venom (BV) is one of the most remarkable natural products that has been a subject of studies since ancient times. Recent studies have shown that *Apis mellifera syriaca* venom possesses antibacterial as well as cytotoxic effects on cancer cell lines. The venom contains a variety of bioactive molecules—mainly melittin (MEL) and phospholipase A2 (PLA2), as well as other compounds that are not well characterized. In this work, we continue the biological characterization of *A. mellifera syriaca* venom by testing its anticoagulant effect on human plasma using the prothrombin time (PT) test, as well as assessing its proteolytic activity. In addition, the cytotoxicity of the crude venom—and of its two main components, MEL and PLA2—was tested on HeLa cancer cell lines for the first time. The results obtained showed the capacity of *A. mellifera syriaca* venom to increase clotting time, thereby proving its anticoagulant effect. Moreover, the venom did not demonstrate a significant proteolytic activity unless administrated at concentrations ≥ 5 mg/mL. Finally, we showed that crude *A. mellifera syriaca* venom, along with MEL, exhibit a strong in vitro cytotoxic effect on HeLa cancer cell lines, even at low concentrations. In summary, our findings could serve as a basis for the development of new natural-based drug candidates in the therapeutic field.

## 1. Introduction

Bees, one of the most remarkable social insects, are widespread, with more than 4000 species comprising both domestic and wild bees. Western honeybees (*Apis mellifera*) are eusocial insects found in Europe, Africa, North America, and the Middle Eastern region [1,2]; they are considered to be the primary pollinators of agricultural and horticultural crops [3].

*Apis mellifera syriaca*, also known as the “Syrian bee”, is a subspecies of the Middle Eastern *Apis mellifera* bee (Lebanon, Syria, Jordan, Palestine, Iraq) [4]. This Middle Eastern insect presents several ecological characteristics, such as its adaptation to the hot and dry Mediterranean climate; it also presents a higher resistance to pests and pathogens than European bees, with more honey production, making it an interesting venomous insect to study [5].

Natural products are known to be a rich source of pharmaceutical molecules with minor side effects. Many studies have highlighted the importance of bee venom (BV), which has become one of the most examined natural products in recent years [6]. BV is a complex blend of bioactive molecules such as melittin (MEL), phospholipase A2 (PLA2), apamin, and hyaluronidase [7,8,9]. The use of BV for medicinal purposes—also known as apitherapy, or particularly bee venom therapy (BVT)—can be traced back thousands of years [10,11]. For example, BV has been used to treat diseases such as rheumatoid arthritis, neurodegenerative diseases, etc. Moreover, BV exerts an anticancer effect against several types of cancer cell lines by inducing apoptosis and necrosis [12,13,14].

MEL is the most abundant peptide in crude *A. mellifera* venom, corresponding to 40–60% of its composition; it exerts many biological activities, including antibacterial, antifungal, and anticancer activities [15,16]. It can disrupt the cell membrane by inserting itself across the phospholipid bilayer, leading to the formation of either transient or permanent pores [17]. Moreover, MEL is a lytic peptide that works by destabilizing the membranes of erythrocytes, resulting in hemolysis [18]. MEL is considered to be the most studied hymenopteran compound, as it possesses many therapeutic applications, including anticancer, anti-inflammatory and antiviral activities [19]. As a matter of fact, MEL was used in a phase II clinical trial study consisting of a group of patients suffering from chronic hepatitis B virus (HBV). The novel biotechnology, which is based on the RNA interference (RNAi) mechanism to induce efficient knockdown of target genes, employs MEL as an endosomolytic biopeptide in order to facilitate the delivery of short interfering RNA (siRNA) conjugates to the desired hepatocytes [20].

Bee venom-phospholipase A2 (Bv-PLA2), a hydrolytic enzyme, belongs to the group III sPLA2 enzymes [14]; it is considered to be the most toxic enzyme in BV, and accounts for 12–15% of the crude BV’s dry weight [21]. Studies have shown that the activity of BV-PLA2 can be induced by MEL during the erythrocyte lysis process as well as the cytotoxic activity against cancer cells, proving the existence of a synergistic action between the two biomolecules [22,23]. Furthermore, the synergistic action between MEL and Bv-PLA2 was also demonstrated in a recent study, where the combination of Bv-PLA2 with MEL improved the inhibition extent of membrane-bound *Escherichia coli* F1F0-ATPAse [15].

Thrombophilia, also known as hypercoagulable state, is when the blood tends to clot too much [24]. This can result in deep-vein thrombosis or arterial thrombosis, which can increase the risk of strokes, severe leg pain, heart attacks, and even amputations [25]. The number of people dying from thrombosis is increasing each year. At present, thrombosis is considered to be the among the leading causes of mortality—especially in the Western world [26].

Cancer-associated thrombosis—more specifically, venous thromboembolism (VTE)—is one of the most serious and most common causes of death in patients with malignancy [27]. Studies have shown that conventional cancer treatments, such as chemotherapy, can play a major role in increasing the risk of developing VTE in cancer patients in comparison to non-cancer patients [28]. Although many guidelines recommend the use of prophylactic low-molecular-weight heparin and direct oral anticoagulants for the treatment of cancer-associated VTE, their advantage is restricted by bleeding complications in cancer patients, mainly occurring at gastrointestinal sites [29,30]. As a result, the design of innovative anticancer drug candidates that also possess anticoagulant effects is highly relevant, and could imply the use of natural products.

In this context, and in order to improve the therapeutic value of the *A. mellifera syriaca* venom, we continue the biological characterization of this natural extract which previous studies demonstrated its capacity to inhibit the proliferation of antibiotic-resistant bacterial strains [4]. Moreover, it has been shown that *A. mellifera syriaca* venom possesses a strong dose-dependent blocking effect on *Escherichia coli* F_1_F_0_-ATPase (with an IC_50_% higher than that obtained with the reference inhibitor of this enzyme) [15]. Furthermore, the venom has shown a considerable cytotoxic effect on different cancer cell lines, such as MCF-7 and HCT116 cell lines [4,23]. In this work, we evaluate for the first time the effects of *A. mellifera syriaca* venom on blood coagulation, as well as its proteolytic activity. Additionally, the cytotoxic effect of the crude venom was analyzed in parallel with its two main components—MEL and PLA2—on HeLa cancer cell lines.

## 2. Results

### 2.1. Effect of A. mellifera syriaca Venom on Human Plasma Coagulation

**Figure 1:**
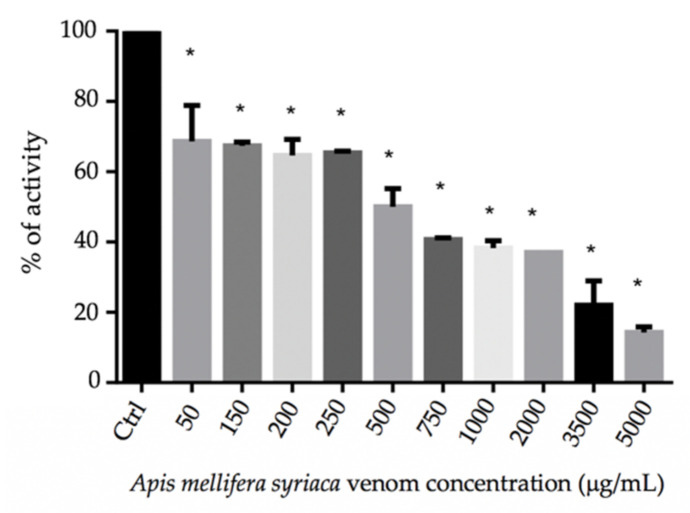
Percentage of plasma activity as a function of the concentration of *A. mellifera syriaca* venom. The results represent the mean ± SD of three independent experiments. Statistically compared with untreated cells; * *p* ˂ 0.0001.

In order to evaluate the effect of *A. mellifera syriaca* venom on blood clotting, the prothrombin time (PT) test was performed. This test measures the activity of extrinsic coagulation factors—in particular, coagulation factor II, also known as prothrombin, which is a biological precursor of thrombin [31]. The results obtained reveal that *A. mellifera syriaca* venom shows an anticoagulant activity from a concentration as low as 50 µg/mL (Table 1, Figure 1). In fact, for a concentration of 50 µg/mL, the time necessary for the blood to clot is 17.2 s with 68.7% plasma activity (see Section 4). The clotting time increases proportionally with the increase in the venom’s concentration, reaching a maximum time of 62.3 s at a concentration of 5000 µg/mL. In contrast, the activity of the plasma decreases with the increase in *A. mellifera syriaca* venom, achieving a minimum activity of 14.3% at a concentration of 5000 µg/mL (Table 1, Figure 1).

It is essential to note that the three parameters evaluated in this test are time (in seconds), which measures how long it takes for the blood to clot, and whose normal value is generally 13.3 s; the activity (in %) of the plasma, which in the normal case should be 100%; and the INR (international normalized ratio), which represents the PT of the blood sample, and which in the normal case should be equivalent to 1. For the positive control, heparin (33 USP units/mL blood) was used—a drug that acts as an anticoagulant, and whose administration has shown significantly low activity (<10%) as well as a considerably high clotting time of 90 s.

Regarding the presence of blood clots in the human plasma tested, we first noticed their formation as fine filaments using a venom concentration of 1 mg/mL. However, their presence became more evident for venom concentrations ranging from 50 µg/mL to up to 750 µg/mL (Table 1).

### 2.2. Proteolytic Effect of A. mellifera syriaca Venom

Honeybee venom is known to contain very few proteases [32] and peptidases [33]. Hence, in order to ensure that the proteolytic effect of the *A. mellifera syriaca* venom is not significant unless administrated at relatively high doses, the following proteolytic assay was performed. The skim milk agar plate method was originally used to assess the capacity of bacteria to degrade proteins. This capacity is due to the presence of proteases—enzymes that degrade proteins. The casein present in milk has a whitish, opaque appearance in the middle. If the sample possesses proteolytic activity, the enzymes will degrade the casein, leading to the loss of the white agar appearance around the deposits. The activity is therefore relative to the diameter of the area of clarification [34].

**Figure 2:**
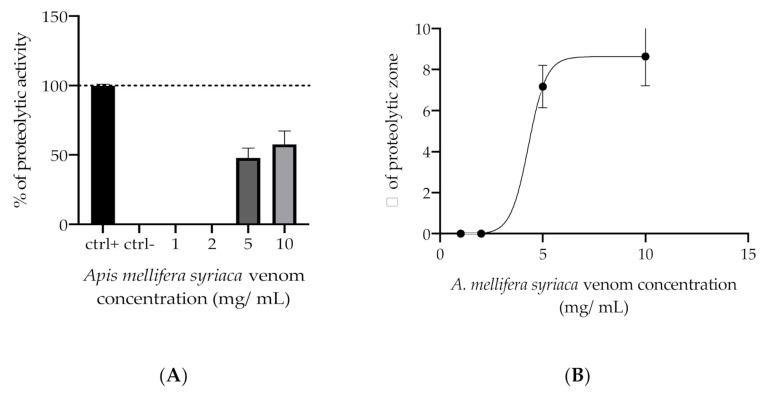
Proteolytic activity of *A. mellifera syriaca* venom, showing a dose-dependent effect at the concentrations being tested: (**A**) The proteolytic effect percentage of venom at the concentrations tested is presented in comparison with the positive control, inducing a percentage effect of 100% (positive control/pepsin), and the negative control, which has a percentage effect of 0% (negative control/H_2_O). (**B**) The dose–response curve of the venom is presented relative to the diameter of the proteolytic zone observed in the Petri dish, and corresponding to the degradation of casein by the *A. mellifera syriaca* venom. The results represent the mean ± SD of three independent experiments.

Four different concentrations of *A. mellifera syriaca* venom were used (1, 2.5, 5, and 10 mg/mL), along with a pepsin solution used as a positive control. The results show that the proteolytic effect is only observed at considerable concentration of the venom (Figure 2A). Effectively, a minimum concentration of 5 mg/mL of the venom is needed in order to trigger the degradation of casein. This translates to the smallest thinning diameter, which is around 6 mm. The activity slightly increases to reach 7 mm of diameter at a concentration of 10 mg/mL (Figure 2B). The slight proteolytic activity observed could be associated with the presence of a serine protease enzyme in the *Apis mellifera* venom [31]. The presence of this enzyme could enhance the distribution of the venom in the blood, leading to better circulation when used in vivo, without the intolerable consequence of protein digestion.

### 2.3. Cytotoxicity of A. mellifera syriaca Venom and of Its Two Main Components—MEL and PLA2—On HeLa Cancer Cell Lines

**Figure 3:**
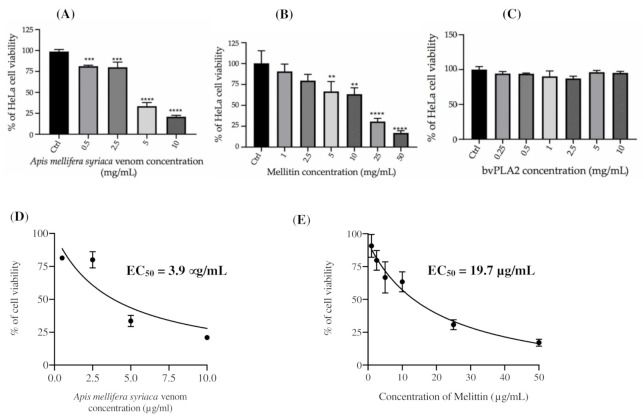
Cell viability of HeLa cancer cells, as measured by MTT test after treatment with increased concentrations of (**A**) *A. mellifera syriaca* venom, (**B**) MEL, and (**C**) Bv-PLA2. The results represent the mean ± SD of three independent experiments. Statistically significant compared with untreated cells (control): ** p < 0.01, *** p < 0.001, **** *p* < 0.0001. (**D**) Curve for MTT assay showing the EC_50_ value and % of cell viability as a function of log concentration of *A. mellifera syriaca* venom. The obtained EC_50_ of venom on HeLa cells was 3.9 µg/mL. (**E**) Curve for MTT assay showing the EC_50_ value and % of cell viability as a function of log concentration of melittin. The obtained EC_50_ of melittin on HeLa cancer cells was 19.7 µg/mL.

After demonstrating the cytotoxic effect of *A. mellifera syriaca* venom on HCT116 cells in our previous work [23], we aimed to expand our research by testing the effect of the same venom on different cancer cell lines. Accordingly, four concentrations of *A. mellifera syriaca* venom were tested on HeLa cancer cells. The results were expressed as the percentage of cell viability in comparison to untreated control cells with 100% viability. The test used to calculate the percentage of cell viability was the MTT test. For a concentration of 0.5 µg/mL, 81% of cells remained viable. This percentage significantly decreased with the increase in the venom concentration, reaching a minimum cell viability of 21% (Figure 3A). These results indicate the presence of a significant cytotoxic effect of *A. mellifera syriaca* venom on HeLa cells, with an EC_50_ of 3.9 μg/mL (Figure 3D).

Furthermore, cells were exposed to increasing concentrations of MEL (1, 2.5, 5, 10, 25, 50 µg/mL) for 24 h. The results obtained showed the presence of a strong cytotoxic effect of MEL against HeLa cancer cells. MEL inhibited the cell viability in a dose-dependent manner. A concentration of 5 µg/mL of MEL was able to induce a significant cytotoxic effect compared to untreated cells. The maximum cytotoxic effect was shown at a concentration of 50 µg/mL, with only 17% viability (Figure 3B). The observed EC_50_ of MEL was 19.7 µg/mL (Figure 3E).

Finally, six concentrations of Bv-PLA2 were tested (0.25, 0.5, 1, 2.5, 5, 10 µg/mL). The results demonstrated that Bv-PLA2 alone does not exert any cytotoxic effect against HeLa cells, and no dose-dependent effect was observed (Figure 3C).

Altogether, these findings confirm the cytotoxic effect of *A. mellifera syriaca* venom on HeLa cancer cells in vitro. Additionally, the results support the hypothesis of our previous work, which suggests the importance of MEL in conferring the BV’s anticancer properties [23]. Moreover, by comparing the EC_50_ values of BV, MEL, and Bv-PLA2 with those of our previous work on human colon cancer cells [23], we found that MEL is more effective on human colon cancer cells (EC_50_ = 14.05 µg/mL) than on HeLa cells (EC_50_ = 19.7 µg/mL). In contrast, BV and Bv-PLA2 did not show a significant difference between the two types of cancer cells. This finding supports our first hypothesis regarding the mechanism of action of MEL against cancer cells not only by acting on different signaling pathways, but also by disturbing the cell membrane. However, this should be validated by further experimental studies.

## 3. Discussion

In this work, we demonstrated the anticoagulant effect of *A. mellifera syriaca* venom using the prothrombin time (PT) test. In normal conditions, the activity of the plasma should range between 70% and 100% in order for the blood to clot. When this activity drops below 70%, it indicates hypoprothrombinemia. Our results show that at a concentration of 50 μg/mL, the *A. mellifera syriaca* venom was able to decrease the human plasma activity to 68%, and in parallel to increase the clotting time to up to 17 s, indicating the presence of an anticoagulant effect. Moreover, we found that the effect of the crude venom on blood coagulation is dose-dependent. In fact, for a concentration of 5000 µg/mL of *A. mellifera syriaca* venom, the plasma activity drastically decreased, reaching as low as 14%; in contrast, the clotting time increased to 62 s. Plasma diluted in ultrapure water was used as a negative control, since the venom is dissolved in water and the values obtained are the same as the reference value. For the positive control, heparin—a drug that acts as an anticoagulant—was used, and its administration showed a significantly low activity (<10%) as well as a significantly high clotting time of 90 s. In sum, these findings confirm the anticoagulant effect of *A. mellifera syriaca* venom. Previous work done by Accary et al. in 2014 studied the effect of *Montivipera bornmuelleri* snake venom on blood coagulation using the same PT test. The results demonstrated that from a concentration of 3 μg/mL the snake venom can act as an anticoagulant. The same study showed that the anticoagulant effect of the snake’s venom is likely due to the presence of enzymes with anticoagulant activity, such as PLA2 [35]. Other studies have shown that *A. mellifera* venom from Iran contains anticoagulant factors that can increase the clotting time (prothrombin time), which is in total agreement with our findings [36]. Likewise, the same study demonstrated that MEL and PLA2 (which are also present in *A. mellifera syriaca* venom) are mainly responsible for this anticoagulant effect. This study supports another finding that showed that PLA2 from the venom of Egyptian honeybee *Apis mellifera lamarckii* exerts anticoagulant activities [37].

Moreover, it is known that the mechanism of coagulation involves activation, adhesion, and aggregation of platelets, along with deposition and maturation of fibrin [38]. A series of reactions are involved, in which inactive serine protease enzymes and their glycoproteins are activated, and can catalyze the next reaction in the cascade, leading to the coagulation of the blood [38]. Therefore, the mechanism of action of BV can be due to its effect on certain coagulation factors—such as inhibition of the serine protease enzymes—or it might have a direct effect on the platelets.

Proteases, and specifically serine proteases, play a major role in coagulopathies, and can act by driving the thrombotic and thrombolytic cascades [39]. A study conducted by Choo et al. in 2010 showed that serine protease from bumblebees’ Bi-VSP acts as a prothrombin activator [32]. Given that the venom showed a significant anticoagulant effect, it is crucial to confirm that *Apis mellifera syriaca* venom—at the concentrations that demonstrated anticoagulant effects—does not possess a proteolytic activity that might contribute to the acceleration of blood coagulation, due the presence of its serine proteases. In this work, the proteolytic activity of *A. mellifera syriaca* venom was assessed in order to ensure that the effect is not significant unless the venom is administrated at concentrations ≥ 5 mg/mL. The results obtained are in accordance with the literature, given that the proteases and peptidases present in the BV only represent a very small proportion of its total composition. Specifically, proteases and peptidases only constitute 1% of the BV’s total weight [8]. This explains why the proteolytic effect of the *A. mellifera syriaca* venom was only present at concentrations ≥ 5 mg/mL. Additionally, the crude BV contains serine protease inhibitors, which could also explain the absence of any proteolytic activity at moderate concentrations [40].

Finally, we demonstrated for the first time the cytotoxic effect of *A. mellifera syriaca* venom on HeLa cancer cell lines. The cytotoxic effect was significant even at low concentrations (0.5 µg/mL) of the crude venom. For a concentration of 5 µg/mL, the proliferation of more than half of HeLa cells was inhibited. In addition, many studies have demonstrated that MEL—the main component of BV—is responsible for the cytotoxic effect of this extract [38,41], which also explains why Bv-PLA2 alone did not show any cytotoxic effect on HeLa cancer cells in our work. Our findings correlate with those of Mansour et al. in 2021, whose in vitro data showed a dose-dependent antiproliferative activity of both BV and MEL on HepG2 cells, with the latter showing the highest cytotoxicity [42]. This is also consistent with our prior findings regarding *A. mellifera syriaca* venom, where MEL was proven to be the compound responsible for the cytotoxic effect on HCT116 human colon cancer cells—especially given its synergistic action with Bv-PLA2 [6].

In fact, the EC_50_ values obtained for the *A. mellifera syriaca* venom and MEL on HeLa cell lines correlate with those observed on HCT116 cells, where they were found to be equal to 3.14 µg/mL for the *A. mellifera syriaca* venom [6]. In contrast, MEL was more effective on HCT116 human colon cancer cells, with an EC_50_ = 14.05 µg/mL [23], than on HeLa cells, with an EC_50_ = 19.7 µg/mL. Our results also correlate with previous studies that demonstrated the anticancer effects of BV against other types of cancer cells, such as lung cancer cells (with an IC_50_ = 2 µg/mL) [43] and ovarian cancer cells (with an IC_50_ = 1.5 µg/mL) [44]. However, MEL showed some variability in the IC_50_ values between different cancer cell types, and even between the cell strains of the same type of carcinoma. For example, the IC_50_ of MEL in ovarian cancer cells was equal to 3.8 µg/mL [44], whereas it was equal to 4.06 µg/mL and 9.24 µg/mL in MHCC97-H and MHCC97-L hepatocellular carcinomas, respectively. Interestingly, these two cell strains have different Rac1 expression levels, indicating that melittin’s anti-HCC effect depends on Rac1 [45]. The main mechanism of action by which both BV and MEL act as anticancer agents is via inducing apoptosis in tumor cells. For example, MEL can trigger both extrinsic and intrinsic pathways, by increasing the expression levels of many pro-apoptotic and apoptotic mediators, such as cytochrome c, protein 53 (p53), Bcl-2-associated X protein (Bax), caspase-3, caspase-9, and different types of death receptors [11,12,46]. In addition, it suppresses the growth factor receptor activation in breast cancer cell lines [41]. It has been also demonstrated that BV can increase the expression of DR3, which induces the apoptosis of lung cancer cells [43]. Moreover, it has been proven that MEL activates the death receptors and inhibits the JAK2L/STAT3 pathway, leading to the inhibition of ovarian cancer cell growth [44]; it can also decrease the expression levels of p-AKT and ERK1/2, and suppress the Rac1-dependent pathway [45].

Despite the wide range of therapeutic applications of both BV and MEL, their unwanted toxicity might limit their clinical development. Hence, many scientists aim to develop new approaches that decrease the toxicity of both BV and MEL. For example, it was demonstrated that loading BV on nanofungal chitosan increased its anti-cancer bioactivity against cervical carcinoma (HeLa) cells by inducing apoptosis in a time- and dose-dependent manner [47].

Finally, the synergistic action of both MEL and PLA2 has long been assessed via chemical processes, such as the hydrolysis of phospholipid monolayers [48,49]. As for the possible existence of a synergistic action between the BV’s two main biomolecules in biological processes, we showed in our latest work that when MEL and Bv-PLA2 were combined together, a potential cytotoxic synergistic effect was observed, and translated into the significant inhibition of the HCT116 cells’ proliferation. Hence, we speculate that a similar synergistic effect between MEL and Bv-PLA2 should also be present for HeLa cancer cells. The mechanism of action is probably the same as that reported by Mufson et al.—MEL enters the phospholipid bilayers and exhibits surfactant activity [50]. The association between MEL and the cellular membranes results in (1) a disturbance of the acyl groups of phospholipids, (2) increased phospholipid susceptibility to hydrolysis by PL, and (3) increased synthesis of PG from the arachidonic acid released from the phospholipids.

Altogether, our results support previous findings on the cytotoxic effects of BV and MEL on different types of cancer cells. Moreover, our study offers better insights on the specificity of MEL’s mode of action against different types of cancer cells.

## 4. Materials and Methods

### 4.1. Chemicals and Reagents

First, 0.5 g of crude bee venom were collected from four beehives of healthy local bees—*A. mellifera syriaca* (Lebanese subspecies) which are found in Matn in Mount Lebanon—by using the electroshock method Then, this venom was freeze-dried and stored at −20 °C. The standard MEL and PLA2 were obtained from LATOXAN—a French laboratory specializing in animal toxins and poisonous animals.

### 4.2. Cell Culture

Human cervical cancer cells (HeLa) were obtained from the American Type Culture Collection (ATCC). Cells were cultured in Dulbecco’s modified Eagle’s medium (DMEM) supplemented with 10% heat-inactivated fetal bovine serum (FBS) and incubated at 37 °C in a humidified atmosphere with 5% CO_2_.

### 4.3. Prothrombin Time (PT) Test

The effect of *A. mellifera syriaca* venom on blood coagulation was studied using the PT (prothrombin time) test [51]. The test was performed in a certified hematology laboratory, using the “STA Compact Max 2” hemostatic analyzer (Bruxelles, Belgium). Freshly collected blood from healthy volunteers who had not taken any medication for at least 2 weeks before sampling was collected in 3.2% sodium citrate tubes and used directly. The samples were centrifuged for 5 min at 4400 rpm, and the plasma was collected. Ten milligrams of *A. mellifera syriaca* venom initial solution was used to achieve the desired BV concentrations to be tested (50, 150, 200, 250, 500, 750, 1000, 2000, 3500, and 5000 μg/mL). Then, 100 μL of each concentrated BV solution was added to 1 mL of plasma solution. As for the negative control, 100 μL of ultrapure water was added to 1 mL of plasma solution. For the positive control, 100 μL of heparin solution was added to 1 mL of plasma solution. The PT test was performed on 1 mL of the plasma incubated with the neoplastic reagent for 1 min at room temperature. Coagulation was initiated by the addition of 0.025 M calcium chloride CaCl_2_. The results were expressed as the time required for the formation of coagulation. The positive and negative controls were used to evaluate the results obtained in the presence of the venom.

### 4.4. Proteolytic Effect

The proteolytic activity of *A. mellifera syriaca* venom was evaluated on milk agar plates. Thus, 2.8 g of Mueller Hinton agar was dissolved in 100 mL of distilled water; the mixture was brought to a boil, and then autoclaved at 121 °C for 20 min. Next, the mixture was cooled, and 11 mL of skimmed and pasteurized milk was added aseptically when its temperature reached 45–50 °C. The mixture was later poured into Petri dishes, which could be used once the medium solidifies. After solidification, 6 mm diameter wells were dug and loaded with 25 μL of the solutions to be tested. The gearboxes were observed for the appearance of clear zones after incubation for 24 h at room temperature.

### 4.5. Cytotoxic Activity Assay on HeLa Cancer Cells

To investigate the venom’s cytotoxicity, the MTT viability test was used [52]. Initial solutions of 1 mg/mL of *A. mellifera syriaca* crude venom, MEL, and PLA2 (Bv-PLA2) were prepared and filtered using a 0.2 µm sterile syringe filter. Cells were cultured in DMEM culture medium until confluence. A 24-well plate was used. 1 mL of sample at the different concentrations was deposited in each well. Triplicate copies were made for the HeLa cells. This plate was incubated at 37 °C for 24 h. After incubation, a volume of 10 μL of MTT was added to each well. This step was performed in the dark, as MTT is photosensitive. The plate was stirred and then incubated at 37 °C for 1 h. The medium was then removed, and 1 mL of DMSO was added to each well in order to solubilize formazan crystals. Absorbance quantification was read at 570 nm.

### 4.6. Statistical Analysis

The results were obtained using one-way ANOVA with Bonferroni’s multiple comparison test, using the GraphPad Prism software; they were presented as the mean ± SD of at least three independent experiments. Statistical significance was defined as * *p* < 0.05, ** *p* < 0.01, and *** *p* < 0.001 compared to untreated cells.

## 5. Conclusions

In this work, we demonstrated that the venom obtained from *A. mellifera syriaca* bees (Lebanese subspecies) possesses an anticoagulant effect. In addition, we showed that the venom’s proteolytic effect is insignificant unless administrated at high concentrations. Finally, we revealed the significant cytotoxic effect of *A. mellifera syriaca* venom as well as its main biopeptide (MEL) on HeLa cancer cells. Notably, we confirmed that Bv-PLA2 does not exert any cytotoxic activity when administrated alone. The biological proprieties of the venom of *A. mellifera syriaca* obtained in this work concerning, on the one hand, the presence of an anticoagulant effect on human plasma and a cytotoxic potential against HeLa cells, and on the other hand, the absence of a significant proteolytic activity, serve as a basis for the possible use of *A. mellifera syriaca* venom for the treatment of human diseases.

## Figures and Tables

**Table 1:** PT test values for different *A. mellifera syriaca* venom concentrations.

*Apis mellifera syriaca* Venom Concentration (mg/mL)	Time (s)	INR	Activity (%)	Description
Reference value	13.3	1	100	
Negative control	13.3	1	100	
Positive control	90.1	>6	<10	
5000	62.3	4.9	14.3	No clots were formed
3500	45.4	3.6	22	No clots were formed
2000	27.2	2.1	37	Clots were formed as small filaments
1000	26.3	2.1	38.3	Idem
750	25.1	1.9	40.6	Idem
500	21.3	1.6	50	Idem
250	17.5	1.3	65.3	Clots were formed partially
200	17.8	1.3	64.7	Idem
150	17.2	1.3	67.3	Idem
50	17.2	1.3	68.7	Idem

## Data Availability

Not applicable.
